# Real-Time Stress Experiences and Physiological and Psychological Responses Among LGBTQ+ Young Adults: Findings from the Stress and Heart Pilot Study

**DOI:** 10.3390/s26123872

**Published:** 2026-06-18

**Authors:** Hee-Jin Jun, Kang-Hyuk Lee, Dulce Urueta Tapia, Jerel P. Calzo, Heather L. Corliss

**Affiliations:** 1 San Diego State University Research Foundation, San Diego, CA 92182, USA; klee17@sdsu.edu (K.-H.L.); duruetatapia@sdsu.edu (D.U.T.); jcalzo@sdsu.edu (J.P.C.); hcorliss@sdsu.edu (H.L.C.); 2 School of Public Health, San Diego State University, San Diego, CA 92182, USA; 3 Institute for Behavioral and Community Health, San Diego State University, San Diego, CA 92182, USA

**Keywords:** LGBTQ+ young adults, minority stress, ecological momentary assessment, wearable sensors, heart rate variability, physiological stress, positive affect, negative affect, discrimination, microaggressions

## Abstract

LGBTQ+ individuals experience disparities in cardiovascular health, but little is known about how daily minority and general stress affect physiological and psychological responses in real-world settings. Twenty LGBTQ+ young adults aged 18–27 completed a 14-day exploratory pilot study using ecological momentary assessment (EMA) with four daily smartphone surveys and continuous smartwatch-based sensor monitoring. This study is among the first to combine EMA with wearable sensor data to capture autonomic stress responses to minority stressors in naturalistic settings. Outcomes included a physiological stress score derived from heart rate variability during the 60 min before each EMA completion, as well as positive and negative affect (PA and NA). Four stress measures, Everyday Discrimination Scale (EDS), Sexual Orientation Microaggression Inventory Short Form (SOMI-SF), EMA of stressful events (EMA-SE), and current perceived stress (CPS), and a combined variable (COMB) were examined. In mixed-effects within-person models, all stress measures showed trends in the expected direction, with higher physiological stress scores, lower PA, and higher NA, though these varied in magnitude and statistical significance. SOMI-SF showed the strongest association with physiological stress, while general stress measures showed stronger associations with affect. These preliminary findings suggest that LGBTQ+-specific and general stressors may differentially engage physiological and psychological response systems; however, caution is warranted given the small sample size.

## 1. Introduction

Lesbian, gay, bisexual, transgender, nonbinary, and other sexual and gender minority (LGBTQ+) individuals experience disproportionately poorer mental and cardiovascular health compared with heterosexual and cisgender populations [1,2]. These disparities emerge in adolescence and continue during young adulthood, developmental periods characterized by heightened exposure to stress and the early accumulation of cardiovascular risk [3,4,5]. Cardiovascular disease remains the leading cause of death in the United States [6], and mental health conditions are a leading cause of morbidity [7], underscoring the importance of identifying the early physiological and psychological mechanisms through which stress contributes to cardiovascular and mental health vulnerability among LGBTQ+ young adults. A large body of research has established stress exposure as a fundamental pathway to both poor mental and cardiovascular health, with chronic stress contributing to dysregulation across autonomic, inflammatory, and metabolic systems that simultaneously undermine psychological and cardiovascular health [8,9,10].

### 1.1. Stress, Autonomic Functioning, and Cardiovascular Risk

Exposure to stress activates a coordinated biological response, commonly referred to as the fight-or-flight response. This response is characterized by the release of stress hormones such as cortisol and catecholamines and by alterations in autonomic nervous system (ANS) activity aimed at maintaining homeostasis [11]. While these responses are adaptive in the short term, prolonged or repeated stress exposure can result in sustained dysregulation of physiological systems [9,12]. Stress-related disruptions in ANS functioning represent a key pathway linking stress exposure to adverse cardiovascular outcomes, including an increased risk of hypertension, myocardial infarction, and stroke [12]. Persistent stress has been associated with reduced heart rate variability (HRV), an indicator of diminished parasympathetic regulation and impaired physiological recovery from stress [11,13]. Lower HRV has been prospectively linked to increased cardiovascular morbidity and mortality across populations [14,15], highlighting HRV as an important early marker of cardiovascular vulnerability. Among LGBTQ+ individuals, chronic exposure to stigma-related stress has been associated with poorer cardiovascular profiles [16], including dysregulation of autonomic functioning [17].

### 1.2. Stress, Affect, and Mental Health

Chronic stress has been linked with poorer mental health [8,9,10], including an elevated risk of depression [18], anxiety [19], post-traumatic stress disorder (PTSD) [20], and substance use disorders [21]. Stress disrupts emotional regulation by heightening amygdala reactivity and prefrontal inhibition, fostering rumination, worry, and hypervigilance [22,23]. Stress also impairs cognitive processes, including attention, memory, and executive functioning, which can lead to maladaptive coping behaviors such as substance use [8]. Studies using daily diary and EMA methods have found that daily LGBTQ+-specific minority stress and microaggressions predict same-day negative affect and next-day affective disturbance in gay, bisexual, and sexual minority adolescent populations [24,25]. Positive affect is also disrupted by stress exposure, with both chronic and acute stressors linked to reduced positive emotional states and lower well-being, and with daily stressors shown to diminish positive affect above and beyond their effects on negative affect [26,27].

### 1.3. The Additive Burden of Minority Stress

LGBTQ+ individuals experience elevated stress across multiple domains. In addition to general life stressors faced by the broader population, they experience stressors specific to stigma, discrimination, and marginalization related to minority sexual orientation and gender identity. Minority stress theory conceptualizes these identity-related stressors as chronic and socially patterned, arising from experiences such as victimization, microaggressions, and social exclusion [28,29,30]. Microaggressions are subtle, repeated slights that communicate hostility toward marginalized identities, and have been associated with an increased risk of depression, anxiety, and PTSD [31,32]. Over time, these distal stressors can give rise to proximal stress processes, including internalized stigma, expectations of rejection, and identity concealment, which further amplify stress exposure [29,30,33]. The Psychological Mediation Framework extends this perspective by positing that minority stress affects health through the dysregulation of underlying psychological processes, including emotion dysregulation, social and interpersonal problems, and negative cognitive styles, which elevate the risk of depression, anxiety, and substance use [30]. This framework has since been extended to explain LGBTQ+ physical health disparities, with minority stress linked to cardiovascular health and physiological dysregulation [8,9,14,34,35]. Empirical studies have linked LGBTQ+ minority stress to poorer cardiovascular profiles [36,37]; however, the mechanisms underlying these associations are not yet fully understood.

### 1.4. Gaps in the Literature

Despite strong theoretical and empirical support, most research on the health impacts of minority stress among LGBTQ+ populations has relied on cross-sectional or retrospective survey designs [36,37,38]. These approaches limit ecological validity and obscure the dynamic, moment-to-moment processes through which minority stress may influence physiological functioning in daily life. Although studies using daily diary or ecological momentary assessment (EMA) methods to examine minority stress are increasing, only a small number have incorporated objective, continuous physiological measures from wearable sensors. EMA is a research method that collects data from individuals in their natural environments in real time [39]. Pairing EMA with real-time, continuous physiological monitoring has the potential to identify mechanisms underlying LGBTQ+ disparities in mental and cardiovascular health. To our knowledge, no published studies have examined real-time associations between minority stress experiences and autonomic nervous system functioning using integrated EMA and wearable sensor data among LGBTQ+ young adults while also capturing general stress experiences occurring during the same period.

### 1.5. The Present Study

To address these scientific gaps, the current study integrates real-time assessments of daily stress with concurrent measurements of physiological and psychological responses among LGBTQ+ young adults. Using intensive longitudinal data collected in naturalistic settings with wearable sensors, this study examines both minority-specific and general stress experiences and their associations with physiological stress regulation and psychological responses. Physiological stress was indexed by the Garmin stress score, a validated measure derived from HRV data [40]. Psychological responses were assessed as positive affect (PA) and negative affect (NA). Specifically, we examined whether (1) stress experiences are associated with physiological stress responses, (2) stress experiences are associated with psychological stress responses, and (3) these associations differ by type of stress experience, specifically whether minority-specific and general stress experiences show different patterns of association with physiological and psychological outcomes.

## 2. Materials and Methods

### 2.1. Study Design and Data Collection

This pilot study used a 14-day intensive longitudinal design combining EMA with wearable sensor data collected from April through November 2022. The feasibility and acceptability of this pilot study have been reported previously [41]. The current analysis focused on examining associations between stress experiences and psychological and physiological responses in the sample. Participants were LGBTQ+ young adults aged 18–30 years recruited from the San Diego area. To increase the probability that participants would report exposure to minority stress during the study period, a prescreening online survey was used to identify and select individuals who reported experiencing minority stress related to their LGBTQ+ status at least “sometimes” on two or more occasions in the past year, based on items adapted from the Everyday Discrimination Scale [42]. Recruitment used both online and in-person methods, including flyers distributed through LGBTQ+ community organizations. After a review of early EMA responses from the first 6 participants, additional stress measures were added to better capture stress experiences relevant to LGBTQ+ young adults; therefore, analyses involving those measures were limited to the last 14 participants enrolled. Additional details regarding study procedures have been reported previously [41].

At the in-person baseline and training session, research staff delivered informed consent, administered the baseline survey, and reviewed the 14-day study procedures with participants. Research staff also instructed participants on how to install the mEMA Sense smartphone application (ilumivu Inc., Asheville, NC, USA) on their personal iOS or Android devices and trained them to pair the app with the Garmin Vivosmart 4 (Garmin International, Inc., Olathe, KS, USA) wearable sensor via Bluetooth. Participants also completed a test EMA survey to confirm proper functioning of the app-based survey system and wearable connection. Example screens from the mEMA app interface have been shown elsewhere [41].

During the 14-day study period, participants completed four EMA surveys and one end-of-day survey per day using an interval-contingent schedule tailored to their daily routines. Prompting generally began within 1 h after waking and continued until bedtime, with approximately 3-h intervals between prompts. To accommodate work, school, and other fixed commitments, intervals could be adjusted within a range of 2 to 4.5 h. Across participants, the first daily EMA prompt occurred between 6:00 a.m. and 11:45 a.m., and the fourth prompt occurred between 4:00 p.m. and 9:00 p.m. The mEMA app delivered four EMA surveys and one end-of-day survey at five participant-specific times each day and issued up to five reminder notifications at 5-min intervals if a survey was not opened. Surveys expired after 30 min and were recorded as nonresponses if not completed within that window. Participants also received daily reminder text messages to support adherence to the survey and wearable protocol. A follow-up Zoom meeting (Zoom Communications, Inc., San Jose, CA, USA) was conducted approximately 2 days after study initiation when needed to troubleshoot technical or scheduling issues. EMA surveys were designed to be brief, typically required less than 3 min to complete, to enhance the response rate and reduce participant burden [43].

### 2.2. Wearable Sensor Data Acquisition and Processing

The Garmin Vivosmart 4 is a wrist-worn wearable device that uses photoplethysmography (PPG) to continuously and unobtrusively measure physiological data. The Garmin Vivosmart 4 was selected because it was compatible with the mEMA Sense platform (ilumivu Inc.), which allowed direct integration of EMA and wearable sensor data in a single protocol, consistent with its use in other mEMA-based studies [44]. As a commercially available device with published validation evidence for the Garmin stress score, it was also feasible to implement within the budget constraints of this pilot study. The wristband is a convenient wearable device for monitoring physiological parameters compared with a chest-worn electrocardiogram (ECG) device, even though the latter provides more accurate HRV data. The Garmin Vivosmart 4 was paired with the mEMA Sense app via Bluetooth to support synchronization of wearable and EMA data. Participants were asked to put on the device each morning and wear it throughout the day for the 14-day study period. They were instructed to charge the device each night or as needed, keep it on during showering and exercise, and wear it relatively tightly to improve heart rate detection. To support continuous data collection, participants were also asked to keep Bluetooth enabled on their phones, keep the mEMA Sense app open throughout the study period, and ensure that heart rate, interbeat interval (IBI), and stress data remained enabled in the app.

The physiological variable analyzed in this study was the Garmin stress score, an HRV-derived, algorithmically processed stress index [45], a measure that has recently been evaluated in the Garmin Vivosmart 4 [40]. Although the Garmin stress score is HRV-derived, it is an algorithmically processed composite index and should not be interpreted as equivalent to a direct HRV measure or a laboratory-grade ECG-based autonomic assessment. According to Firstbeat documentation, this stress metric is derived from beat-to-beat heart rate data (RR intervals) after artifact filtering and resampling, followed by second-by-second analysis and segmentation into physiologically stationary periods. The algorithm first identifies physical activity and recovery from physical activity, and it evaluates stress only during the remaining non-exercise-related segments. Because the algorithm excludes exercise and immediate post-exercise recovery, the stress signal is not continuous and may contain gaps during physically active periods [40,46]. Rosenbach et al. [40] assessed the Garmin-calculated stress score (GSS) against ECG recordings by a Polar H10 chest strap in a laboratory setting and found that GSS exhibited significant differences between stress and rest. They also found that GSS correlated significantly with heart rate, root mean square of the successive differences (RMSSD), a time-domain measure of HRV, and the ratio of standard deviation 2/standard deviation 1 (SD2/SD1), a non-linear HRV metric that measures autonomic balance. These findings indicate that GSS is a valid indicator of physiological stress.

The daily EMA prompts yielded 56 planned prompts per participant across the 14-day data collection period. EMA responses were linked to wearable data using time stamps. For each completed EMA survey, the primary corresponding HRV-derived stress score was derived from the 60-min period preceding EMA submission. The optimal aggregation window for HRV-derived stress measures depends on the psychological phenomenon of interest and the research question [47]. Across different EMA studies, the time-aggregated HRV measure has typically ranged from 5 min [48] to 30 min [49,50,51] to 60 min [52]. In naturalistic settings where the exact timing of stressors relative to EMA prompts is difficult to know with certainty, a longer (e.g., 60 min) aggregation window is preferable because autonomic recovery following psychological stress can take up to 60 min or even longer [50,53,54]. Consistent with this logic, a 2-week EMA study that combined HRV monitoring with EMA found that the HRV-derived stress score averaged over the 60 min preceding the EMA prompt significantly predicted perceived stress levels [52]. To evaluate the selection of the primary 60-min window, we examined EMA reports of the timing of the stress experience from the final 14 participants for whom these data were available. Approximately 51% of the reported stress experiences occurred within 60 min of the EMA prompt, and 25% occurred within the preceding 1–2 h, for a cumulative 77% within 2 h. Because autonomic recovery from psychological stress may extend 60 min or longer after the stressor [54,55], the 60-min window captures the physiological traces of a meaningful proportion of reported stressors. Given that these methods are relatively novel in the context of physiological responses to everyday stress experiences, we also examined the average stress score derived from 30-min and 120-min windows preceding EMA submission as sensitivity analyses. If no stress observation was available during the pre-EMA aggregate window, the sensor value for that EMA observation was treated as missing. Missing sensor data due to nonwear, charging, synchronization failure, or the lack of an algorithm-derived stress value were left as missing rather than imputed.

### 2.3. Measures

#### 2.3.1. Primary Outcomes

As noted previously, the primary physiological outcome was the Garmin Vivosmart 4 stress score, calculated from HRV and averaged over the 60-min period preceding each participant’s completion of an EMA survey. The continuous stress score ranges from 0 to 100, with higher values indicating greater physiological stress.

The primary psychological outcomes were positive affect (PA) and negative affect (NA), which were assessed using an adapted version of the validated Positive and Negative Affect Schedule (PANAS) [56]. To ensure that the EMA survey was appropriate for the LGBTQ+ young adult population, the original items were reviewed in focus groups. Based on qualitative feedback, a final subset of five PA and six NA items was selected for use in the EMA surveys. PA and NA scores were calculated separately as the means of their respective items. Details of the selected items are provided in Appendix A, Table A1.

#### 2.3.2. Primary Predictors

Stress experiences were assessed at each EMA prompt using three event-based measures and a single item measuring current perceived stress. Each of the four measures was coded as a separate variable, and an additional combined indicator was created to classify prompts with any reported stress experience across the four stress measures versus no reported stress experience.

The first event-based measure, everyday discrimination, was assessed using an adapted version of the Everyday Discrimination Scale (EDS) [42], which is designed to capture experiences of unfair treatment in daily life based on various identities and statuses (e.g., race/ethnicity, sexual orientation, gender, gender identity, age, weight). The original scale demonstrated strong internal consistency (Cronbach’s α = 0.88) and construct validity across physical and mental health outcomes in a large adult sample [42]. For the present study, we used a 13-item version adapted for EMA administration and minority populations from several sources [57,58,59,60,61]. At each EMA prompt, participants were asked whether they had experienced any discriminatory events since the last prompt and to select all that applied from the 13-item list, including an “other” option. If a discriminatory event was reported, follow-up questions assessed whether the event was related to specific statuses (e.g., sexual orientation, gender identity, gender, race/ethnicity, mental health, physical health, appearance), as well as the timing and location of the event. For analysis, any report of an event on the EDS was coded as positive for an EDS stress experience regardless of attribution.

After data collection from the first six participants, we reviewed the EMA responses and concluded that stress experiences specific to LGBTQ+ young adults were not fully captured by the adapted EDS alone. Therefore, we added two additional event-based measures and one item assessing perceived stress to the EMA survey and administered these new measures to the final 14 participants.

One measure added captured LGBTQ+-related microaggressions assessed using the Sexual Orientation Microaggression Inventory Short Form (SOMI-SF) [62,63], which was adapted for this study. The SOMI-SF is a validated 8-item measure demonstrating high internal consistency (α > 0.80) and significant associations with LGBTQ+-based victimization, depression, and anxiety across three diverse samples of sexual minority youth. For the present study, we adapted the SOMI-SF by adding one additional item assessing the use of incorrect pronouns to capture gender identity-specific microaggressions and changing the response options from a 5-point Likert scale to binary yes/no response to align with the EMA format. At each EMA prompt, participants indicated whether someone had said something similar to the listed statements about themselves or about other LGBTQ+ people and selected all that applied. Example items include “being told not to act so gay, butch, or queer”; “hearing that being LGBTQ+ is just a phase”; and “hearing someone use the wrong personal pronoun”. If endorsed, a follow-up question assessed the timing of the event [62].

General stress was also added and assessed using an Ecological Momentary Assessment of Stressful Events (EMA-SE), a brief measure of momentary stress experiences developed by the Science of Behavior Change initiative [64] and designed to minimize retrospective recall bias by capturing stressful events close to the time of occurrence. Participants were asked whether any other stressful event had occurred since the last prompt that negatively affected them. If they responded yes, they selected the type of event (e.g., argument or conflict, financial event, home-related event, work-related event, health event, event affecting others, traffic or transportation event, other) and reported the timing and perceived stressfulness of the event.

Current perceived stress (CPS) was assessed using a single-item measure: “At the moment, I feel stressed”, with responses ranging from “not at all” to “extremely” [65]. Single-item stress measures have demonstrated good test–retest reliability (ICC 0.66–0.74) [66], strong correlations with Cohen’s Perceived Stress Scale (r = 0.62) [67], and satisfactory construct validity for group-level analysis [68]. In the context of EMA, validity is further supported by theoretically expected within-person associations rather than traditional test–retest reliability—that is, a valid stress measure should show higher scores at moments when stressful things happen to a person than at moments when they do not [69]. CPS was originally assessed on an ordinal Likert-type scale and was dichotomized for the primary analyses to align with the other stress measures and facilitate comparison across measures. Responses of “moderately”, “quite a bit”, or “extremely” were categorized as higher stress, whereas responses of “not at all” or “a little” were categorized as lower stress.

Finally, a combined measure of stress experiences was calculated (COMB), which included any reports of a stressful experience captured by the EDS, SOMI-SF, EMA-SE, and CPS versus no report on any of these measures.

#### 2.3.3. Demographic Variables

Demographic characteristics were assessed via the baseline survey using questions commonly used in health surveys. Age was collected as a continuous variable. Race and ethnicity were assessed by first asking participants about their ethnicity and then about their race, with response options including American Indian or Alaska Native, Asian, Black or African American, Middle Eastern or North African, Native Hawaiian or other Pacific Islander, White, and some other race. Sexual orientation was categorized as lesbian/gay, bisexual, pansexual, or queer. Gender identity was categorized as cisgender man, cisgender woman, transgender man, transgender woman, or non-binary. Marital status was categorized as single, living with a partner, or not living with a partner. Employment status was categorized as employed full-time, employed part-time, or student. Annual income was classified into five categories: under $20,000, $20,000 to $39,999, $40,000 to $59,999, $60,000 to $79,999, or ‘prefer not to answer’.

### 2.4. Analysis

Descriptive statistics were calculated at two distinct levels of analysis. First, participant-level characteristics were summarized using means for variables treated as continuous and frequencies for variables treated as categorical to describe the study sample. Second, outcome variables were summarized at the prompt level to characterize the longitudinal data collected across all observations.

Mixed-effects regression models were employed to evaluate associations between stress experiences and both physiological and psychological outcomes across repeated EMA observations within individuals [70,71]. Each model was bivariate and unadjusted, with one stress measure entered at a time as the sole predictor. Given the pilot nature of the study, the limited sample size, the limited number of matched EMA-sensor observations, and the smaller analytic sample for measures added later in the study, all analyses were exploratory and hypothesis-generating, intended to provide preliminary estimates of associations and to inform the design of a future fully powered study. For these reasons, adjusted mixed-effects models and covariate-based sensitivity analyses were not performed. Including multiple time-varying and person-level covariates in this dataset could have increased model complexity relative to the available data, with a greater risk of imprecise or unstable estimates [72,73,74]. To account for individual differences in baseline levels of the outcome variables, a random intercept was specified for each participant. Stress experiences were operationalized both as discrete individual measures and as a binary indicator representing a combination of all reported stress versus no stress. All models used a first-order autoregressive covariance structure with heterogeneous variances to address potential serial correlation and variance instability. Statistical significance was determined using a two-sided alpha level of 0.05, although qualitative interpretation also considered the magnitude of the associations and the confidence intervals given the small sample size of the pilot. Model assumptions were evaluated using residual diagnostics from the fitted mixed-effects models. Visual inspection of the residual Q-Q plots suggested no substantial departure from normality for the stress-score models and only moderate upper-tail deviation for the PA and NA models. Because linear mixed-effects models are generally robust to mild-to-moderate departures from normality, these diagnostics did not indicate substantial violations of model assumptions.

Wearable sensor data availability was evaluated for the primary 60-min pre-EMA window. Of the 1001 completed EMA prompts, 673 (67.2%) had corresponding wearable stress score data. For analyses involving the later-added stress measures (SOMI-SF, EMA-SE, CPS, and COMB), 421 matched EMA-sensor observations were available within the 14-participant subsample. To characterize potential differences between prompts with and without matched sensor data, we compared stress experience reporting rates by sensor data availability. Stress experiences were reported more often at matched than unmatched prompts across all measures. For example, any stress incident was reported at 52.97% of matched prompts compared with 31.65% of unmatched prompts. These differences were statistically significant, with small-to-modest effect sizes (Cramer’s V = 0.09–0.21). We also compared sensor data availability by day of the week. Missing sensor data were somewhat more common on weekends than on weekdays (32.58% vs. 27.46%), although this difference was not statistically significant (*p* = 0.071, Cramer’s V = 0.054).

Sensitivity analyses were conducted to evaluate the impact of variable coding decisions. Specifically, CPS was additionally modeled using its original ordinal response scale, and COMB was alternatively modeled as a count of reported stress measures. Results from these sensitivity analyses are presented in Supplementary Table S2.

#### 2.4.1. Physiological Outcomes

For physiological outcomes, analyses examined whether stress experiences were associated with higher levels of physiological stress scores. To test whether the findings were consistent across different time frames, sensitivity analyses were conducted using alternative stress aggregation windows. These windows included the 30-, 60-, and 120-min periods prior to each EMA survey.

#### 2.4.2. Psychological Outcomes

For psychological outcomes, positive affect (PA) and negative affect (NA), which were assessed via PANAS [56], were examined in relation to stress experiences using the same mixed-effects regression framework. Because PANAS outcomes were reported contemporaneously at each EMA prompt, no time-window aggregation or sensitivity analyses were conducted.

## 3. Results

### 3.1. Participant Characteristics

Participants (*N* = 20) had a mean age of 21.7 years (SD: 2.59; range: 18–27). As shown in Table 1, the sample was diverse in race/ethnicity, sexual orientation, and gender identity. Participants identified as non-Hispanic White (45%), non-Hispanic Black (5%), Hispanic (25%), and non-Hispanic Asian (10%). Three participants (15%) identified as multiracial, including one as non-Hispanic Black and Asian and two as non-Hispanic White and Asian. Sexual orientation was reported as lesbian/gay (40%), bisexual (10%), pansexual (20%), and queer (30%). Gender identity was reported as cisgender man (20%), cisgender woman (30%), transwoman/transfeminine (5%), transman/transmasculine (10%), and non-binary (35%). Most participants were single (65%), 45% were students, and 60% reported an annual income under US $20,000 (Table 1).

### 3.2. Prompt-Level Observations

Across 20 participants, 1120 EMA prompts were scheduled (56 per participant), of which 1001 were completed. Corresponding wearable data in the 60-min pre-EMA window were available for 673 completed EMA prompts. As described in the Methods, the Garmin stress score is generated only for algorithm-eligible segments rather than continuously for all minutes of wear time, which contributed to the smaller number of matched EMA-sensor observations compared with completed EMA reports. Among the final 14 participants, 421 matched EMA-sensor observations were available for analyses involving stress experience measures added later in the study.

### 3.3. Descriptive Statistics for Physiological and Psychological Outcomes

Table 2 presents prompt-level descriptive statistics for physiological stress scores, PA, and NA by each stress experience measure. In the 20-participant analysis, discrimination experiences reported via the EDS were associated descriptively with higher mean physiological stress score, lower mean PA, and higher mean NA compared with periods when no EDS discrimination was reported. Among the 104 EDS-reported discrimination events, 66 (63%) were attributed to sexual orientation, gender identity, or gender, either alone or in combination with other attributions. In the 14-participant analyses, reports of stress experiences measured through the adapted SOMI-SF, EMA-SE, and CPS generally showed a similar pattern, with higher mean physiological stress score, lower mean PA, and higher mean NA in periods when a stress experience was reported compared with periods when no stress experience was reported.

### 3.4. Physiological Outcomes: Stress Score

**Figure 1:**
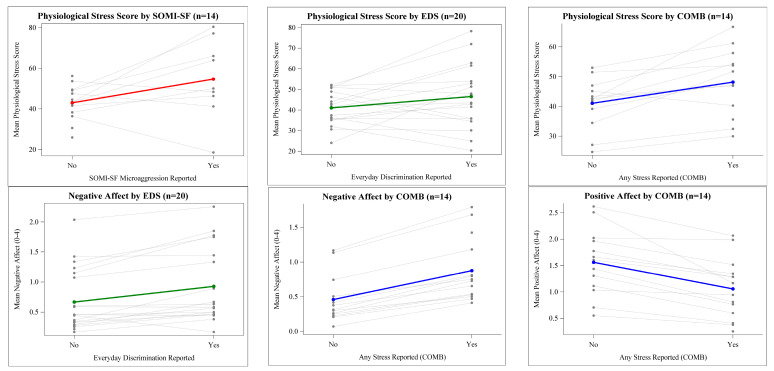
Individual-level mean physiological and psychological responses by stress experience indicators among LGBTQ+ young adults. Note: SOMI-SF = Adapted Sexual Orientation Microaggression Inventory–Short Form; EDS = Everyday Discrimination Scale; COMB = combined stress experience variable; NA = negative affect; PA = positive affect. The top panels present mean physiological stress scores based on the 60-min pre-EMA window by SOMI-SF, EDS, and COMB. The bottom panels present NA by EDS and COMB and PA by COMB. Gray lines represent individual participants, and colored lines represent group means for each predictor: red = SOMI-SF, green = EDS, and blue = COMB. SOMI-SF and COMB analyses include 14 participants; EDS analyses include 20 participants.

Table 3 shows the associations between stress experiences and physiological stress scores and psychological responses (PA and NA). EDS analyses were based on all 20 participants, whereas analyses for SOMI-SF, EMA-SE, CPS, and COMB were based on the 14 participants who completed those measures. For the physiological stress score, SOMI-SF (β = 8.16, 95% CI: 1.02–15.31; *p* = 0.025) and COMB (β = 5.93, 95% CI: 1.53–10.34; *p* = 0.008) were associated with higher stress scores, while EMA-SE and CPS showed positive but statistically nonsignificant associations. In the 20-participant analyses, EDS was associated with a higher physiological stress score but did not reach statistical significance (β = 4.06, 95% CI: −0.66–8.79; *p* = 0.091). Figure 1 illustrates both group means and variability in responses across participants. The top panels show higher mean physiological stress scores when a stress experience was reported than when none was reported across all three indicators, with considerable variability in individual-level patterns.

### 3.5. Psychological Outcomes: Positive Affect and Negative Affect

For psychological outcomes, the pattern was clearer for NA than for PA and stronger for general stress measures (EMA-SE, CPS) than for the LGBTQ+-specific stress measure. In the 20-participant analysis, EDS was associated with higher NA (estimate = 0.22, 95% CI 0.13 to 0.30; *p* < 0.0001), but not with PA (estimate = −0.06, 95% CI −0.17 to 0.05; *p* = 0.308). In the 14-participant analyses, SOMI-SF was not associated with either PA or NA. In contrast, the general stress measures showed a consistent pattern across both affective outcomes. EMA-SE, CPS, and COMB were each associated with lower PA and higher NA (all *p*s < 0.0001). Figure 1 also displays individual-level mean NA by EDS and COMB and mean PA by COMB, showing within-person patterns alongside group means. The bottom panels show higher mean NA when a stress experience was reported for both EDS and COMB and lower mean PA when any stress experience was reported for COMB, with substantial variability across individuals.

### 3.6. Supplementary and Sensitivity Analyses

Standardized effect-size estimates and variance-explained measures are presented in Supplementary Table S1. The standardized estimates were consistent with the unstandardized estimates reported in Table 3. For the physiological stress score, SOMI-SF showed the largest standardized association (standardized β = 0.37), followed by COMB (β = 0.27), EMA-SE (β = 0.24), EDS (β = 0.19), and CPS (β = 0.16). For psychological outcomes, general stress measures showed the largest standardized associations. CPS, EMA-SE, and the combined stress measure were associated with lower PA (standardized β range: −0.61 to −0.35) and higher NA (standardized β range: 0.51 to 0.68). In contrast, SOMI-SF showed comparatively weaker standardized associations with PA and NA. Marginal R^2^ values were small across models, as expected for single-predictor mixed-effects models in clustered EMA data, whereas conditional R^2^ values were larger, particularly for PA and NA, indicating that participant-level random effects accounted for an important share of the total explained variance.

Sensitivity analyses using alternative coding strategies also yielded results consistent with the primary analyses. When CPS was modeled using its original ordinal response scale, associations were in the same direction as the dichotomized CPS models for the physiological stress score (β = 1.22, *p* = 0.273), NA (β = 0.24, *p* < 0.001), and PA (β = −0.29, *p* < 0.001). Similarly, when the combined stress variable was modeled as a count of reported stress measures, associations were consistent with the binary combined stress models for the physiological stress score (β = 3.60, *p* = 0.006), NA (β = 0.23, *p* < 0.001), and PA (β = −0.23, *p* < 0.001). Full results are presented in Supplementary Table S2.

Sensitivity analyses using 30-, 60-, and 120-min pre-EMA windows showed generally similar directions of association across windows, although the magnitudes of the estimates varied. For the combined stress measure, the estimated associations with the physiological stress score were similar for the 30-min and 60-min windows and smaller, but still positive, for the 120-min window. For SOMI-SF, the estimate was largest for the 60-min window, whereas estimates for EDS, EMA-SE, and CPS were smaller and less consistent across windows. Full results are presented in Supplementary Table S3.

## 4. Discussion

### 4.1. Summary of Findings

This 14-day pilot study, which integrated EMA and wearable sensor data, provides preliminary evidence that daily stress experiences are associated with both physiological and psychological responses among LGBTQ+ young adults. Given the limited sample size and the exploratory, hypothesis-generating aims of the analyses, these findings should be interpreted as preliminary estimates of association intended to inform the design of a future fully powered study. The observed patterns further suggest that associations between stressor type and outcomes may differ across physiological and psychological domains. For the physiological outcome, the LGBTQ+-specific stress measure, the adapted Sexual Orientation Microaggression Inventory (SOMI-SF), was significantly associated with higher physiological stress scores. In contrast, the two general stress measures, Current Perceived Stress (CPS) and the EMA of Stressful Events (EMA-SE), as well as the Everyday Discrimination Scale (EDS), showed weaker positive associations that were not statistically significant. This pattern is consistent with findings from a 7-day daily diary study of 121 sexual and gender minority young adults that examined both general daily stressors and LGBT-specific stressors in relation to a different physiological outcome [75]. In that study, participants who reported more LGBT-specific stressors had higher cortisol levels at waking and 45 min after waking, even after accounting for general stressors and covariates. By contrast, general stressors were associated only with higher cortisol 12 h after waking. Together, these findings suggest that minority stress may have a stronger and more distinctive association with physiological stress processes than general stress.

A different pattern emerged for the psychological outcomes. The two general stress measures were more strongly and significantly associated with both lower positive affect (PA) and higher negative affect (NA), whereas SOMI-SF showed weaker, non-significant associations with both affective outcomes. Associations involving EDS were mixed. The EDS showed the weakest association with PA of all the stress measures and was more strongly associated with NA than SOMI-SF, but less strongly associated than CPS and EMA-SE. Across measures, stress experiences were more consistently associated with NA than with PA. Whether LGBTQ+-specific stressors and general stressors have different relationships with affective outcomes remains unclear and will require further study, particularly because our review did not identify studies that examined minority stress, general stress, and psychological outcomes together within LGBTQ+ samples. Given the pilot design, small sample size, and varying analytic samples across measures, these findings should be interpreted cautiously and replicated in larger, more diverse samples.

### 4.2. LGBTQ+-Specific Stress Experiences and Physiological and Psychological Responses

The finding that LGBTQ+-specific stressors, measured by the adapted SOMI-SF, were significantly associated with higher physiological stress scores derived from HRV in a real-time naturalistic setting represents a novel contribution of this study and extends prior research on LGBTQ+ minority stress and physiological functioning. A 2024 systematic review of 53 experience-sampling (i.e., EMA or daily diary) studies of everyday LGBTQ+ stress and health published between 2007 and 2022 found that most studies focused on mental and behavioral health outcomes [76]. Only five of the studies reviewed examined physical health outcomes, all of which relied on self-report rather than objective physiological measures, and none used wearable sensors to capture autonomic responses in daily life. In related work, Flentje et al. reviewed 26 studies published through 2018 that examined associations between minority stress and biological outcomes among LGBTQ+ populations and found that most were cross-sectional (65%), with smaller numbers of longitudinal (12%), short-term repeated-measures (15%), and experimental studies (8%) [36]. Importantly, no studies examining HRV were identified. Updating and expanding on this work, Flentje et al. synthesized 59 studies published between 2019 and 2024 among LGBTQ+ populations and found associations between minority stress and a broad range of biological outcomes, including cardiovascular outcomes [37]. Among these, however, the majority used cross-sectional or longitudinal observational designs, 5 used experimental laboratory paradigms and 2 used daily diary methods [37]. Of the studies examining heart rate or HRV [17,77,78] none incorporated EMA or real-time wearable sensor data in naturalistic settings [37].

Consistent with these reviews, the small body of research examining minority stress and physiological responses indexed by HRV among LGBTQ+ populations has been limited to laboratory settings. Keenan et al., using a modified Trier Social Stress Test incorporating sexual orientation- and race-based discrimination among 274 female participants (66.4% Black American, half of whom identified as LGB), found that HRV reactivity to the task varied significantly by sexual orientation and race, with Black LGB women showing larger HRV decreases during the task compared to White LGB women [77]. Huebner et al. randomized 134 LGB adults (51% male, 49% female; ages 18–58) to an anti-gay or pro-gay confederate condition and found that high-frequency HRV remained stable throughout the task in the pro-gay condition but decreased significantly during the task in the anti-gay condition [78]. Paralleling the findings of the current study, this suggests that LGB-specific stressors produce a qualitatively different physiological pattern beyond general social stress. Rosati et al., however, found results in an unexpected direction. Among 19 LGB adults and 20 matched heterosexual participants, they found that compared to heterosexual participants, LGB individuals showed unexpectedly higher resting HRV, a finding that runs counter to the typical association between stress and reduced parasympathetic activity, which is expressed as low HRV [17]. Taken together, these laboratory studies demonstrate that minority stress is associated with distinct autonomic and cardiovascular responses in LGBTQ+ individuals, though the direction and nature of these associations vary across studies. Given that these studies were conducted in the laboratory, it remains unknown whether such associations extend to everyday naturalistic settings.

Laboratory findings that have examined other physiological health indicators beyond HRV also support the detrimental impact of LGBTQ+ stressors. Huebner et al. found that, in addition to reduced high-frequency HRV, participants in the anti-gay condition showed greater increases in heart rate and systolic blood pressure during the task, smaller decreases in systolic blood pressure during recovery, and increases in salivary cortisol from before to shortly after the task [78]. Rosati et al. reported that, compared with heterosexual participants, LGB participants showed higher vascular resistance at rest and a more vascular hemodynamic response during stress tasks [17]. A more vascular hemodynamic response means that the cardiovascular response is driven more by blood vessel constriction, or increased peripheral resistance, than by increases in cardiac output. This pattern was especially pronounced during the LGB-related emotional task. This finding is broadly consistent with our result that LGBTQ+ stressors may be especially detrimental to physiological response. At the same time, LGB participants also showed higher resting HRV, creating a paradoxical pattern in which greater parasympathetic activity and greater vascular resistance co-occurred. The authors interpreted this pattern as potentially reflecting chronic effortful emotion regulation in response to repeated discrimination. Among LGB participants, lower self-reported day-to-day minority stress was associated with a more vascular response during the LGB-related task. The authors suggested that this counterintuitive pattern may reflect coping processes operating outside awareness, drawing on prior work on the internalization of discrimination, rather than indicating that these participants were experiencing less discrimination.

In naturalistic settings outside the laboratory, daily diary studies have linked discrimination experiences to cortisol responses. Seaton and Zeiders, in a daily diary study of Black adults, found that daily racial discrimination experiences were associated with diurnal cortisol patterns, suggesting that discrimination-related stress can affect physiological functioning in everyday life [79]. Among LGBTQ+ populations specifically, two studies have examined associations of minority stress with cortisol. Figueroa et al., in a 7-day daily diary study, of 121 LGBTQ+ young adults aged 18–35 (54.5% female, 82% cisgender, 61% identified as gay, lesbian, or homosexual), found that participants who reported more LGBTQ+-specific stressors across the week had higher waking cortisol, above and beyond the effects of general stressors [75]. This finding broadly aligns with the findings of the current study that LGBTQ+-specific stressors and general stressors differently contribute to physiological response. Cook et al. reported, in a 2-day virtual daily diary study of 20 young gay and bisexual men aged 18–35 from the New York metropolitan area, that greater everyday discrimination was associated with lower mean daily cortisol [80]. The opposite patterns observed across these studies, higher cortisol in Figueroa et al. and lower cortisol in Cook et al., may reflect differences in the stressors assessed, the methods used to measure cortisol, or the study samples. These mixed findings underscore the need for more rigorous research using longer-term, real-world designs.

Taken together, these studies suggest that LGBTQ+-specific stressors may influence multiple physiological systems, including autonomic, vascular, and neuroendocrine stress response systems, but most evidence has come from laboratory-based or short-term naturalistic studies. The current study extends this literature by integrating EMA with continuous wearable sensor data to capture real-time autonomic stress responses to LGBTQ+-specific stressors in participants’ natural daily environments.

An important empirical question in this field is the extent to which LGBTQ+ stressors affect health through direct physiological stress pathways versus indirectly through health behaviors that later influence physical health. Theory and emerging evidence suggest that both pathways likely operate, but that the effects of minority stress are not fully explained by behavior. In a cross-sectional study of 357 sexual- and gender-diverse adults, Chuntova et al. found that both major lifetime discrimination and day-to-day discrimination were independently associated with higher allostatic load, an index of cumulative physiological wear and tear, after controlling for age [81]. These associations were not explained by substance use, sleep quality, or physical activity, suggesting that discrimination may be directly linked to biological dysregulation through stress response systems. Although cross-sectional data cannot establish causality, this finding adds to a growing literature indicating that LGBTQ+ minority stress may contribute to health disparities through direct physiological as well as indirect behavioral mechanisms. Accordingly, reducing LGBTQ+ health disparities will likely require interventions that address structural stressors alongside individual health behaviors and physiological risk processes.

As highlighted in Nicholas & Bresin’s systematic review [76] and subsequent research [82,83], a larger number of experience sampling (i.e., EMA, daily diary) studies have examined the impacts of minority stress on psychological outcomes compared with physiological outcomes. Across 53 experience sampling studies [76], daily minority stressors were linked to higher NA in 27 of 28 studies and lower PA in 10 of 15 studies, demonstrating that minority stress is more consistently associated with NA than with PA. Aligning with the conclusions of this systematic review, a subsequent 28-day daily diary study of 99 cisgender bisexual women found that sexual orientation microaggressions predicted higher same-day NA but not PA [83]. In the current study, we found different associations between minority stress experiences and NA and PA depending on the specific measure. Associations between the LGBTQ+-specific stress measure (adapted SOMI-SF) and both negative affect (higher NA) and positive affect (lower PA) were in the expected direction. The association was stronger for NA than for PA, but neither association was statistically significant, possibly due to the small sample size. Although the Everyday Discrimination Scale (EDS) was not designed exclusively to measure LGBTQ+-specific experiences, in the current sample more than half of the EDS-reported discrimination events were attributed to LGBTQ+ status, making it at least partially a minority stress measure in this context. In our study, EDS was significantly associated with higher NA, while its association with lower PA was weaker and not statistically significant. Replication in larger samples is needed to determine whether SOMI-SF and EDS show real different patterns of affective responses. Prior experience sampling studies examining associations between minority stress and NA and PA among LGBTQ+ populations have been limited to daily diary studies. The current study extends this literature by examining LGBTQ+-specific stress experiences as predictors of momentary PA and NA in real-time naturalistic settings using EMA.

Our study suggests that minority stress experiences may have different real-time associations with physiological and psychological responses. In particular, our measure of LGBTQ+-related minority stress, the adapted SOMI-SF, was more strongly associated with physiological responses than with psychological responses. This finding adds to a growing literature suggesting that minority stress may manifest differently across response systems in daily life. Prior EMA and daily diary research has often emphasized associations between minority stress and psychological distress and affective functioning [24,76], whereas a broader body of work indicates that minority stress can also become biologically embedded and reflected in physiological processes [37,75]. Our findings suggest that the real-time impact of minority stress may be evident in autonomic functioning even when concurrent affective responses are weaker or less apparent. This pattern may reflect heightened physiological vigilance or regulatory demands that is not always captured by brief self-reports of affect. Overall, our results support the view that minority stress can have meaningful immediate physiological correlates and underscore the importance of assessing both psychological and physiological responses in everyday-life studies of minority stress.

### 4.3. General Stress Experiences and Physiological and Psychological Responses

Measures of general stress in our study (EMA-SE and CPS) were positively associated with the physiological stress score, although these associations did not reach statistical significance. This pattern is consistent with the broader EMA literature on general stress and physiological responses in daily life. In a systematic review of 104 EMA studies examining acute stressors and physiological reactions under naturalistic conditions, Weber et al. found that only about half of the reviewed studies reported positive associations between everyday stress exposure and concurrent physiological responses, including cardiovascular outcomes such as blood pressure and heart rate [84]. Similarly, Vaessen et al. reviewed 36 studies comprising 135 analyses of self-reported stress and ambulatory cardiovascular measures collected over at least one day and found that only 35% of analyses showed a significant association in the expected direction [85]. Among cardiovascular measures, frequency-domain HRV showed the most consistent correspondence with self-reported stress, with significant associations observed in 54% of analyses, whereas other cardiovascular measures were considerably less consistent [85]. Taken together, these reviews suggest that subjective stress and concurrent physiological responses do not always align in naturalistic settings. In the current study, it remains unclear whether the non-significant associations reflect a true null relationship or limited statistical power to detect an effect of this magnitude. Larger studies will be needed to clarify this question.

In contrast, both general stress measures showed consistent and statistically significant associations with psychological outcomes. EMA-SE and CPS were each associated with lower positive affect (PA) and higher negative affect (NA) at the same EMA prompt. These findings are consistent with prior work on daily stress and affect. For example, in an 8-day daily diary study of 1517 midlife non-LGBTQ+ adults (mean age = 57.1 years), Rackoff and Newman found that participants reported spending less time in positive affect on days when stressors occurred than on days without stressors, and that greater reductions in PA on stressor days predicted an increased risk for major depressive disorder and anxiety disorders seven years later [86]. The current findings also align with theoretical accounts of PA and NA as partially independent affective systems with different sensitivities to daily stressors. In a systematic review and meta-analysis of 74 EMA and daily diary studies, Boemo et al. found large effect sizes for the association between NA and maladaptive responses to daily experiences, whereas associations involving PA were smaller and more variable [87]. This pattern suggests that NA may respond more directly and strongly to daily stressors, whereas PA may depend more on active engagement with positive aspects of the environment. Consistent with this interpretation, PA has been theorized to operate closer to a stable set point and to be more sensitive to the presence of positive experiences than to the absence of stress, whereas NA tends to rise in response to negative events that demand attention [26].

A review of the literature identified only one experience sampling study that examined general stressors and psychological outcomes in an LGB sample. In that study, Wardecker et al. assessed 98 LGB adults and a comparison sample of 3323 heterosexual adults and found that LGB participants experienced daily stressors more often than heterosexual participants and showed greater same-day NA reactivity to those stressors [88]. LGB participants were also especially likely to report work/school stressors and discrimination, and exploratory analyses suggested that heightened NA reactivity may have been particularly pronounced among bisexual participants. The stressor measure used in that study differs somewhat from ours because it combined both minority-related and general stressors, making it most similar to our broader combined stress measure rather than to EMA-SE or CPS alone. Even so, its findings are broadly consistent with ours, particularly with respect to the association between stress experiences and higher NA. Overall, our findings suggest that general and LGBTQ+-specific stress experiences may show different patterns of association with physiological and psychological response systems, a possibility that warrants further investigation in larger samples.

### 4.4. Methodological Contributions

Prior research on minority stress and physiological outcomes in LGBTQ+ populations has relied predominantly on laboratory paradigms, retrospective surveys, and daily diary methods using salivary biomarkers [36,37]. Laboratory paradigms have established that acute social-evaluative stressors reliably elicit cortisol and HRV responses under controlled conditions [89], but they have well-recognized limitations for studying stress as it unfolds in daily life, because stressors are artificially induced rather than naturally occurring [84]. While daily diary studies have linked everyday stressors and discrimination experiences to salivary cortisol in naturalistic contexts in other populations [79,90], comparable work capturing real-time physiological responses in LGBTQ+ populations remains limited, as discussed in Section 4.2. A systematic review of 104 EMA studies found that salivary cortisol was the most commonly used physiological outcome, with cardiovascular and HRV measures used far less frequently [84]. These limitations leave a gap in understanding how minority stress affects physiological functioning as it actually occurs in everyday environments.

A small but growing body of research has begun combining EMA with wearable physiological sensing to address this gap in the general population. Akbar et al. demonstrated that a Garmin Vivosmart wearable paired with EMA surveys could capture HRV-based stress fluctuations aligned with perceived occupational stress among physicians [91]. Carreiro et al. similarly found that physiological signals from a wrist-worn sensor aligned with EMA-reported stress in adults in treatment for substance use disorders [92]. More recently, Trujillo et al. used a 21-day EMA protocol with a smartphone’s embedded optical sensor to capture heart rate alongside self-reported stress in a large sample of LGB and heterosexual adults, finding that LGB adults recorded higher heart rate compared to heterosexual adults [93]. However, that study compared groups rather than examining within-person associations between specific minority stress events and concurrent physiological responses. A recent systematic review of 11 EMA studies assessing mental health among LGBTQ+ populations found that none of the included studies incorporated wearable sensors to capture physiological outcomes [94]. The current study extends the EMA-plus-wearable methodology to an LGBTQ+ population in the context of minority stress, capturing real-time autonomic responses to naturally occurring stress experiences in participants’ daily lives. In addition, at 14 days, this study was longer than most prior naturalistic studies of minority stress and physiological outcomes in this population. Cook et al. conducted a 2-day virtual daily diary study [80], and Figueroa et al. used a 7-day daily diary design [75]. A longer study period increases the number of observations per person and improves the likelihood of capturing minority stress events as they naturally occur across varied daily contexts.

A second methodological contribution is the use of a comprehensive stress battery designed to capture the full range of stress that LGBTQ+ young adults experience. The study began with the EDS as the sole EMA stress measure for the first six participants. After reviewing early EMA responses, the research team recognized that the EDS alone did not fully capture stress experiences specific to LGBTQ+ young adults. Three additional measures were therefore added for the remaining 14 participants: the SOMI-SF, EMA-SE, and CPS. This battery covered both LGBTQ+-specific and general stress within the same EMA prompt, and collecting all measures from the same participants at the same time made it possible to directly compare how different types of stressors relate to physiological and psychological outcomes within the same sample. Most prior EMA studies in this population have used a single stress measure, which limits the ability to distinguish between stressor types and their downstream effects.

A third contribution relates to the selection of the physiological aggregation window. For the 14 participants who completed the expanded protocol, a timing question was added asking participants to report when the stress experience occurred since the last EMA prompt. This provided some empirical basis for evaluating the 60-min pre-EMA window. Among those who reported a stress experience and provided timing information, approximately 51% occurred within 60 min of the EMA prompt, and a cumulative 77% occurred within 2 h, supporting the 60-min window as a reasonable choice for this sample and context. Sensitivity analyses across 30-, 60-, and 120-min pre-EMA windows showed that associations were generally similar in direction, but the magnitudes of the estimates varied across aggregation periods. The combined stress measure showed relatively consistent estimates at the 30- and 60-min windows and a smaller but still positive estimate at the 120-min window, suggesting attenuation as the physiological aggregation window widened. This pattern supports the 60-min window as a reasonable primary aggregation period while also indicating that temporal alignment between reported stress experiences and physiological responses remains an important consideration for future EMA-sensor studies.

Together, these methodological features provide a foundation for future research on real-time minority stress and physiological health in naturalistic settings, and they demonstrate that combining EMA with wearable sensing is both feasible and informative in LGBTQ+ young adult populations.

### 4.5. Limitations

Several limitations of this pilot study should be acknowledged. First and most importantly, the sample was small, with 20 participants overall and only 14 completing the SOMI-SF and general stress measures. The study was intentionally designed as a pilot to establish feasibility and acceptability and to generate initial estimates of association. The sample size was not powered to detect effects of a given magnitude or to support robust inference about the statistical significance of individual associations. All findings should therefore be treated as initial estimates, and effect sizes and confidence intervals should be regarded as illustrative rather than definitive. Replication in a larger sample is necessary before conclusions can be drawn about the direction or magnitude of any of the reported associations. The sample was also not large enough to support subgroup analyses or tests of interaction by intersecting statuses such as race/ethnicity, gender identity, sexual orientation, or socioeconomic position, and stress responses may differ across these groups. Second, the observational design of this study limits causal conclusions about the direction of associations between stress experiences and physiological or emotional responses. Reverse causality is possible; for example, elevated physiological arousal or negative affect may influence how stressors are perceived or reported, rather than stress experiences necessarily preceding these responses. Although the study used a within-person EMA approach, which provides stronger evidence of individual-level associations than cross-sectional designs, the direction of effects cannot be established. It is possible, for example, that elevated physiological arousal influences subsequent stress appraisals rather than the reverse, or that both directions operate simultaneously. Future studies with larger samples and denser repeated assessments should use cross-lagged or other time-series approaches to better assess temporal ordering and strengthen causal inference.

Third, the SOGI-related stress measures, including the SOMI-SF, were available only for the 14 participants who completed the final version of the EMA protocol, resulting in smaller and potentially less representative analytic samples for those outcomes. The 14 participants who completed these measures were those enrolled after a mid-study protocol revision, and they may differ from the earlier 20-participant sample in ways that are difficult to assess with such small numbers. Full details of the study protocol, including the sequence of changes made, are described elsewhere [41].

Fourth, physiological stress was assessed using the Garmin Vivosmart 4, a wrist-worn consumer-grade device that uses photoplethysmography (PPG) rather than electrocardiography (ECG). Wrist-based PPG is more susceptible to motion artifacts and provides lower-fidelity HRV data than chest-based ECG, which remains the gold standard for HRV assessment. Data sparsity is a recognized challenge in ambulatory wrist-PPG research. Juarascio et al., in a free-living EMA study using a validated wrist-worn PPG sensor, reported that only 55–58% of HRV data remained usable after quality control, and many time-domain features could not be computed due to insufficient signal continuity, a pattern comparable to the algorithm-eligible segment constraints encountered in the current study [51]. These limitations may have weakened the observed associations in the current study by increasing measurement error in the physiological stress estimate and reducing the number of usable matched EMA-sensor observations, thereby lowering precision and potentially attenuating the strength of the associations. In addition, the physiological stress score was generated by a proprietary Firstbeat algorithm, which limits transparency and independent replication of the exact computational steps. Rosenbach et al. validated the Garmin stress score against ECG-based recordings and found it to be a valid indicator of physiological stress, which supports its use here [40]. However, findings based on this device should be interpreted with awareness that measurement precision is lower than in laboratory-based or ECG-grade ambulatory studies. Broader reviews of EMA research have found that the association between self-reported stress and wearable-derived cardiovascular measures in daily life is modest and inconsistent even in general populations [84,85], and a recent systematic review found that real-time wearable HRV data in naturalistic settings remain rare in LGBTQ+ populations [37], meaning that interpretive benchmarks for the current findings are limited. These observations reinforce the need to treat wearable physiological signals as one source of information alongside, rather than instead of, concurrent self-report measures.

Fifth, a further limitation concerns the completeness of the wearable sensor data. Matched sensor data were not available for all completed EMA prompts, and the missingness analysis suggested that missing sensor data were not entirely random. The exact cause of each missing stress score could not be determined; plausible contributors include device non-wear, charging, and the absence of an algorithm-derived stress value within the aggregation window. Because missingness may have been partially related to stress experiences, the observed associations with the physiological stress score should be interpreted with additional caution. Future studies should monitor device wear compliance more systematically to support more rigorous evaluation of missingness.

Sixth, a related limitation concerns the 60-min pre-EMA aggregation window. Although participant-reported timing data supported this choice, the estimate came from a small subsample (*n* = 14). The best window for capturing physiological responses to minority stress in daily life is still unknown, and the time between a stress experience and its physiological peak may differ across people, stressor types, and situations. Future research should test and compare different window lengths to address this question.

Seventh, several potential confounders of the observed associations were not measured. Physical activity, alcohol consumption, sleep quality, caffeine intake, medication use, time of day, day in the study, and baseline mental health may affect autonomic functioning and therefore influence the physiological stress score. In this pilot study, some potentially relevant time-varying factors were not collected because of resource constraints and the need to minimize participant burden in an intensive EMA and wearable-sensor protocol. Although the Firstbeat algorithm excludes exercise periods from stress score computation [45], residual confounding from these factors cannot be excluded in a naturalistic free-living design. Future fully powered studies should examine these associations using adjusted multivariable models and sensitivity analyses that incorporate potential time-varying and person-level covariates. Finally, the generalizability of the findings is limited by the use of a prescreened, convenience sample of LGBTQ+ young adults recruited from the San Diego area who had reported prior minority stress exposure. The sample may not be representative of LGBTQ+ individuals with lower levels of minority stress exposure, older adults, or those living in geographic contexts with different social and legal climates.

## 5. Conclusions

This pilot study adds to the growing body of evidence that both minority and general daily stress experiences are linked to physiological and psychological responses in LGBTQ+ young adults. LGBTQ+-specific microaggressions, measured in real time using an adapted version of the SOMI-SF, were associated with higher physiological stress scores derived from wearable HRV data, while general stress experiences were more strongly associated with higher negative affect and lower positive affect. The divergence between stressor types across outcomes suggests that LGBTQ+-specific and general stressors may engage different response systems, a pattern that warrants further investigation in larger samples.

This study is among the first to combine EMA with continuous wearable sensor data to capture real-time autonomic stress responses to minority stressors in naturalistic daily life settings. The findings demonstrate that this approach is feasible in LGBTQ+ young adult populations and can generate meaningful data on the physiological correlates of minority stress as it unfolds in everyday life. Given the small sample and pilot design, these results should be treated as initial estimates rather than definitive conclusions. Replication in larger, diverse samples is needed to establish the validity and generalizability of these associations. Future studies should also examine potential moderators, including race/ethnicity, gender identity, sexual orientation, socioeconomic position, social context, and coping resources, that may shape how minority stress translates into physiological and psychological burden over time, including through interaction analyses.

## Figures and Tables

**Table 1:** Participants’ Demographic Characteristics (*N* = 20).

Characteristics	Mean/*n*	SD/%
Age (mean, years)	21.7	2.59
Race/ethnicity		
Non-Hispanic White	9	45
Non-Hispanic Black	1	5
Non-Hispanic Asian	2	10
Hispanic	5	25
Multi-Racial Non-Hispanic Black and Asian	1	5
Multi-Racial Non-Hispanic Asian and White	2	10
Sexual Orientation		
Lesbian/Gay	8	40
Bisexual	2	10
Pansexual	4	20
Queer	6	30
Gender Identity		
Cisgender man	4	20
Cisgender woman	6	30
Transwoman/Transfeminine	1	5
Transman/Transmasculine	2	10
Non-binary	7	35
Employment status		
Employed Full-time	5	25
Employed Part-time	6	30
Student	9	45
Marital Status		
Single	13	65
Living with a partner	2	10
Not living with a partner	5	25
Income (US $)		
Under $20,000	12	60
$20,000–$40,000	3	15
$40,000–$60,000	2	10
$60,000–$80,000	1	5
Prefer not to answer	2	10

Note: *N* = total sample; *n* = number of participants.

**Table 2:** Prompt-Level Descriptive Statistics for Physiological and Psychological Responses by Stress Experiences.

Stress Experience Measure	Physiological Stress Score (1–100)				Positive Affect (0–4)				Negative Affect (0–4)			
	*n*	Mean	SD	Range	*n*	Mean	SD	Range	*n*	Mean	SD	Range
20 Participants (EMA *n* = 1001)												
Everyday Discrimination Scale (EDS)												
No discrimination reported	577	39.9	20.8	(0.3–98.0)	878	1.31	0.89	(0.0–3.8)	878	0.61	0.60	(0.0–3.0)
Discrimination reported	96	46.4	21.2	(6.1–91.3)	123	0.80	0.92	(0.0–3.6)	123	1.41	0.96	(0.0–3.5)
14 Participants (EMA *n* = 699)												
Sexual Orientation Microaggression Inventory (SOMI-SF)												
No microaggression reported	359	41.8	22.2	(2.3–98.0)	618	1.3	0.9	(0.0–3.4)	618	0.6	0.6	(0.0–3.5)
Microaggression reported	62	50.0	20.9	(6.1–91.3)	81	1.1	0.8	(0.0–3.6)	81	1.0	0.7	(0.0–3.3)
EMA of Stressful Event (EMA-SE)												
No stressful event reported	372	42.1	21.8	(2.3–98.0)	635	1.3	0.9	(0.0–3.6)	635	0.7	0.6	(0.0–3.3)
Stressful event reported	49	50.7	23.9	(6.2–92.4)	64	0.9	0.8	(0.0–3.4)	64	1.0	0.7	(0.0–3.5)
Current Perceived Stress (CPS)												
Not at all to a little	258	42.9	22.5	(2.3–98.0)	468	1.6	0.8	(0.0–3.6)	468	0.5	0.5	(0.0–3.2)
Moderately to extremely	163	43.3	21.8	(2.6–92.4)	231	0.7	0.6	(0.0–2.4)	231	1.2	0.7	(0.0–3.5)
Combined Stress experience (COMB)												
No stress experience reported	198	40.5	22.3	(2.3–98.0)	388	1.6	0.8	(0.0–3.4)	388	0.4	0.4	(0.0–2.3)
Stress experience reported	223	45.3	21.8	(2.6–92.4)	311	0.9	0.7	(0.0–3.6)	311	1.0	0.7	(0.0–3.5)

Note: *n* = number of observations.

**Table 3:** Associations between Stress Experiences and Physiological and Psychological Responses.

	Physiological Outcome			Psychological Outcomes					
	Stress Score			Positive Affect			Negative Affect		
	β	*p*-Value	95% CI	β	*p*-Value	95% CI	β	*p*-Value	95% CI
20 Participants (*n* = 673) *									
Everyday Discrimination Scale									
No discrimination reported	0			0			0		
Discrimination reported	4.06	0.091	(−0.66, 8.79)	−0.06	0.308	(−0.17, 0.05)	0.22	<0.0001	(0.13, 0.30)
14 Participants (*n* = 421) *									
Sexual Orientation Microaggression Inventory (SOMI-SF)									
No microaggression reported	0			0			0		
Microaggression reported	8.16	0.025	(1.02, 15.31)	−0.10	0.231	(−0.27, 0.07)	0.08	0.221	(−0.05, 0.20)
EMA of Stressful Event									
No stressful event reported	0			0			0		
Stressful event reported	5.28	0.104	(−1.08, 11.65)	−0.30	<0.0001	(−0.44, −0.16)	0.38	<0.0001	(0.27, 0.48)
Current Perceived Stress									
Not at all to a little	0			0			0		
Moderately to extremely	3.62	0.149	(−1.31, 8.56)	−0.52	<0.0001	(−0.62, −0.42)	0.44	<0.0001	(0.36, 0.51)
Combined Stress Experience									
No stress experience reported	0			0			0		
Stress experience reported	5.93	0.008	(1.53, 10.34)	−0.31	<0.0001	(−0.41, −0.22)	0.33	<0.0001	(0.26, 0.39)

* Prompt-level number of observations (*n*) varies across predictors and outcomes because of measure availability and missing data. Note: Physiological stress scores ranged from 0 to 100 and were averaged over the 60-min period prior to EMA completion. Positive affect and negative affect were calculated as mean scores ranging from 0 to 4. β = unstandardized mixed effect regression parameter estimates. CI = confidence interval.

## Data Availability

The original contributions presented in this study are included in the article/Supplementary Materials. Further inquiries can be directed to the corresponding authors.

## Supplementary Materials

The following supporting information can be downloaded at: https://www.mdpi.com/article/10.3390/s26123872/s1, Table S1: Standardized effect sizes and variance explained for mixed-effects models of stress experiences and physiological and psychological outcomes; Table S2: Sensitivity analyses: Ordinal CPS and count-based COMB models compared to primary binary models; Table S3: Sensitivity analyses: Mixed-effects model estimates for physiological stress scores across 30-, 60-, and 120-min pre-EMA aggregation windows.

## Abbreviations

The following abbreviations are used in this manuscript:

COMB	combined stress variable indicating endorsement of any of the four stress variables
CPS	current perceived stress
EDS	Everyday Discrimination Scale
EMA	ecological momentary assessment
EMA-SE	EMA of stressful events
HIPAA	Health Insurance Portability and Accountability Act
HRV	heart rate variability
IBI	interbeat interval
IRB	Internal Review Board
LGBTQ+	lesbian, gay, bisexual, transgender, queer, and other diverse sexual and gender identities
NA	negative affect
PA	positive affect
PANAS	Positive and Negative Affect Schedule
PPG	photoplethysmography
SOMI-SF	Sexual Orientation Microaggression Inventory Short Form
TPR	total peripheral resistance

## Appendix A

**Table A1:** Ecological Momentary Assessment (EMA) Questionnaires.

**Mood**	
Affective emotional states (I-PANAS-SF)	
Leading question	Right now I feel… Select all that apply.
Items	Happy, Relaxed, Angry, Annoyed, Anxious, Depressed, Afraid, Confident, Inspired, Hopeful, Bored
Response options	Not at all; A little; Moderately; Quite a bit; Extremely
**Stressful Events**	
Everyday Discrimination Scale	
Leading question	Since your last prompt, have you experienced any of the following discriminatory event? Select all that apply.
Items	Treated with less courtesy or respect than others Treated as if others are afraid of you Treated as if others think you are dishonest or immoral Treated as inferior, less smart/capable than others Insulted or called names (direct or overheard) Received poorer service than others (restaurants, stores, etc.) Treated unfairly by your family Stereotyped or negatively labeled Threatened or harassed Avoided, excluded, or ignored Have your perspective/feelings overlooked Physically or sexually assaulted Other
Response options	Yes; No
Follow-up question	You selected (present their selection). Is it related to any of the following statuses? Select all that apply.
Response options	Sexual orientation; Gender identity; Gender; Race/ethnicity; Mental health; Physical health; Appearance; Weight/body size; Religion; Other (Please describe); None/non-applicable
Follow-up question	When were you treated with (present their selection)?
Response options	0–30 min ago; 30–60 min ago;1–2 h ago; 2–3 h ago; More than 3 h ago
Follow-up question	Where were you treated with (present their selection)?
Response options	In-person; Online/mobile device
Follow-up question	You selected In-person. Please describe where it occurred. Select all that apply.
Response options	Work/school; My home; Other’s home; Community space (neighborhood, store, park, gym, bar, restaurant, etc.); LGBTQ community space; Other place (DMV, hospital/clinic, post-office, police station, etc.)
Follow-up question	You selected online. Please describe where it occurred. Select all that apply.
Response options	Phone call/text message; Email/direct message; Work/school meeting; Social media (Twitter, Instagram, Reddit, etc.); Dating apps, Online gaming; Online social gathering (Zoom, Google Meet, etc.); Other online space
Sexual Orientation Microaggression Inventory (SOMI-SF)	
Leading question	Someone said something similar to the following about you or LGBTQ people. (Select all that apply)
Items	Not to act so gay, butch, queer, etc. You know how gay people are Being LGBTQ is just a phase LGBTQ people don’t face discrimination LGBTQ people overreact when talking about a negative experience related to their sexual orientation or gender identity I don’t mind or have nothing against LGBTQ people, but they shouldn’t be so public or open Being LGBTQ is a sin or immoral Used the wrong personal pronoun
Response options	Yes; No
Follow-up question	When did you hear someone say (present their selection).
Response options	0–30 min ago; 30–60 min ago; 1–2 h ago; 2–3 h ago; More than 3 h ago
Ecological Momentary Assessment of Stressful Events	
Items	Did anything else stressful occur since the last prompt that you haven’t mentioned yet? A stressful event is any event, even a minor one, which negatively affected you.
Response options	Yes; No
Follow-up question	How would you describe the stressful event(s)?
Response options	Argument; conflict; disagreement; Financial event; Home-related event; Work-related event; Health event; Event that happened to others; Traffic or transportation event; Other
Follow-up question	When did the event happen?
Response options	0–30 min ago; 30–60 min ago; 1–2 h ago; 2–3 h ago; More than 3 h ago
Follow-up question	How stressful was the event?
Response options	Not at all; A little; Moderately; Quite a bit; Extremely
General Stress	
Items	At the moment, I feel stressed
Response options	Not at all; A little; Moderately; Quite a bit; Extremely
**Substance Use**	
Alcohol Use	
Leading question	Did you drink alcohol?
Response options	No; Yes
Follow-up question	How many alcoholic drinks did you have? (1 standard drink being one 12 oz. beer/wine cooler, 5 oz. glass of wine, one cocktail, or a shot (1.25 oz.) of hard liquor.)
Response options	1 or less; 2; 3; 4; 5; 6; 7 or more
Follow-up question	What were your reasons/motivations for drinking alcohol? Select all that apply.
Response options	Enjoyment/fun/excitement; Coping with sadness/anger/frustration; Coping with tension/stress/anxiety; Relaxation; To socialize/conform socially/peer pressure; Sexual enhancement; It’s a habit/compulsive use reasons; It’s what was available/easier to get; To feel more comfortable as an LGBTQ individual; To relieve/manage boredom; To manage physical pain; To experiment; Influenced by another substance; Other (Please describe)
E-cigarette	
Leading question	Did you vape or use an e-cigarette device?
Response options	No; Yes
Follow-up question	About how many puffs of an e-cigarette did you take?
Response options	1–10; 11–20; 21–30; 31–40
Follow-up question	What were your reasons/motivations for vaping/using an e-cigarette device? Select all that apply.
Response options	Enjoyment/fun/excitement; Coping with sadness/anger/frustration; Coping with tension/stress/anxiety; Relaxation; To socialize/conform socially/peer pressure; Sexual enhancement; It’s a habit/compulsive use reasons; It’s what was available/easier to get; To feel more comfortable as an LGBTQ individual; To relieve/manage boredom; To manage physical pain; To experiment; Influenced by another substance; Other (Please describe)
Cigarette Smoking	
Leading question	Did you smoke cigarettes?
Response options	No; Yes
Follow-up question	How many cigarettes did you smoke?
Response options	1 or less; 2; 3; 4; 5; 6; 7; 8; 9; 10 or more
Follow-up question	What were your reasons/motivations for smoking cigarettes? Select all that apply.
Response options	Enjoyment/fun/excitement; Coping with sadness/anger/frustration; Coping with tension/stress/anxiety; Relaxation; To socialize/conform socially/peer pressure; Sexual enhancement; It’s a habit/compulsive use reasons; It’s what was available/easier to get; To feel more comfortable as an LGBTQ individual; To relieve/manage boredom; To manage physical pain; To experiment; Influenced by another substance; Other (Please describe)
Marijuana	
Leading question	Did you smoke or consume marijuana/cannabis?
Follow-up question	What were your reasons/motivations for smoking or consuming marijuana/cannabis? Select all that apply.
Response options	Enjoyment/fun/excitement; Coping with sadness/anger/frustration; Coping with tension/stress/anxiety; Relaxation; To socialize/conform socially/peer pressure; Sexual enhancement; It’s a habit/compulsive use reasons; It’s what was available/easier to get; To feel more comfortable as an LGBTQ individual; To relieve/manage boredom; To manage physical pain; To experiment; Influenced by another substance; Other (Please describe)

## References

[1] Bonomo J.A., Luo K., Ramallo J.A. (2024). LGBTQ+ cardiovascular health equity: A brief review. Front. Cardiovasc. Med..

[2] Caceres B.A., Streed C.G. (2021). Cardiovascular health concerns in sexual and gender minority populations. Nat. Rev. Cardiol..

[3] Clark C.J., Borowsky I.W., Salisbury J., Usher J., Spencer R.A., Przedworski J.M., Renner L.M., Fisher C., Everson-Rose S.A. (2015). Disparities in long-term cardiovascular disease risk by sexual identity: The National Longitudinal Study of Adolescent to Adult Health. Prev. Med..

[4] Morgan E., D’Aquila R., Carnethon M.R., Mustanski B. (2019). Cardiovascular disease risk factors are elevated among a cohort of young sexual and gender minorities in Chicago. J. Behav. Med..

[5] Sherman J., Dyar C., McDaniel J., Funderburg N.T., Rose K.M., Gorr M., Morgan E. (2022). Sexual minorities are at elevated risk of cardiovascular disease from a younger age than heterosexuals. J. Behav. Med..

[6] Martin S.S., Aday A.W., Almarzooq Z.I., Anderson C.A.M., Arora P., Avery C.L., Baker-Smith C.M., Barone Gibbs B., Beaton A.Z., Boehme A.K. (2024). 2024 Heart Disease and Stroke Statistics: A Report of US and Global Data From the American Heart Association. Circulation.

[7] Udupa N.S., Twenge J.M., McAllister C., Joiner T.E. (2023). Increases in poor mental health, mental distress, and depression symptoms among U.S. adults, 1993–2020. J. Mood Anxiety Disord..

[8] McEwen B.S. (2007). Physiology and neurobiology of stress and adaptation: Central role of the brain. Physiol. Rev..

[9] McEwen B.S. (2017). Neurobiological and Systemic Effects of Chronic Stress. Chronic Stress.

[10] Slavich G.M., Irwin M.R. (2014). From stress to inflammation and major depressive disorder: A social signal transduction theory of depression. Psychol. Bull..

[11] Kim H.G., Cheon E.J., Bai D.S., Lee Y.H., Koo B.H. (2018). Stress and Heart Rate Variability: A Meta-Analysis and Review of the Literature. Psychiatry Investig..

[12] Vaccarino V., Bremner J.D. (2024). Stress and cardiovascular disease: An update. Nat. Rev. Cardiol..

[13] Kemp A.H., Quintana D.S. (2013). The relationship between mental and physical health: Insights from the study of heart rate variability. Int. J. Psychophysiol..

[14] Thayer J.F., Yamamoto S.S., Brosschot J.F. (2010). The relationship of autonomic imbalance, heart rate variability and cardiovascular disease risk factors. Int. J. Cardiol..

[15] Jarczok M.N., Weimer K., Braun C., Williams D.P., Thayer J.F., Gündel H.O., Balint E.M. (2022). Heart rate variability in the prediction of mortality: A systematic review and meta-analysis of healthy and patient populations. Neurosci. Biobehav. Rev..

[16] Mereish E.H., Goldstein C.M. (2020). Minority Stress and Cardiovascular Disease Risk Among Sexual Minorities: Mediating Effects of Sense of Mastery. Int. J. Behav. Med..

[17] Rosati F., Williams D.P., Juster R.P., Thayer J.F., Ottaviani C., Baiocco R. (2021). The Cardiovascular Conundrum in Ethnic and Sexual Minorities: A Potential Biomarker of Constant Coping With Discrimination. Front. Neurosci..

[18] Hammen C. (2005). Stress and depression. Annu. Rev. Clin. Psychol..

[19] Daviu N., Bruchas M.R., Moghaddam B., Sandi C., Beyeler A. (2019). Neurobiological links between stress and anxiety. Neurobiol. Stress.

[20] Yehuda R., Hoge C.W., McFarlane A.C., Vermetten E., Lanius R.A., Nievergelt C.M., Hobfoll S.E., Koenen K.C., Neylan T.C., Hyman S.E. (2015). Post-traumatic stress disorder. Nat. Rev. Dis. Primer.

[21] Koob G.F., Schulkin J. (2019). Addiction and stress: An allostatic view. Neurosci. Biobehav. Rev..

[22] Gerin W., Zawadzki M.J., Brosschot J.F., Thayer J.F., Christenfeld N.J., Campbell T.S., Smyth J.M. (2012). Rumination as a mediator of chronic stress effects on hypertension: A causal model. Int. J. Hypertens..

[23] Hosseini-Kamkar N., Varvani Farahani M., Nikolic M., Stewart K., Goldsmith S., Soltaninejad M., Rajabli R., Lowe C., Nicholson A.A., Morton J.B. (2023). Adverse Life Experiences and Brain Function: A Meta-Analysis of Functional Magnetic Resonance Imaging Findings. JAMA Netw. Open.

[24] Eldahan A.I., Pachankis J.E., Jonathon Rendina H., Ventuneac A., Grov C., Parsons J.T. (2016). Daily minority stress and affect among gay and bisexual men: A 30-day diary study. J. Affect. Disord..

[25] Mereish E.H., Miranda R., Liu Y., Hawthorne D.J. (2021). A daily diary study of minority stress and negative and positive affect among racially diverse sexual minority adolescents. J. Couns. Psychol..

[26] Zautra A.J., Affleck G.G., Tennen H., Reich J.W., Davis M.C. (2005). Dynamic approaches to emotions and stress in everyday life: Bolger and Zuckerman reloaded with positive as well as negative affects. J. Personal..

[27] Sin N.L., Almeida D.M. (2018). Daily Positive Experiences and Health: Biobehavioral Pathways and Resilience to Daily Stress. The Oxford Handbook of Integrative Health Science.

[28] Meyer I.H. (2003). Prejudice as stress: Conceptual and measurement problems. Am. J. Public Health.

[29] Meyer I.H. (1995). Minority stress and mental health in gay men. J. Health Social. Behav..

[30] Hatzenbuehler M.L. (2009). How does sexual minority stigma “get under the skin”? A psychological mediation framework. Psychol. Bull..

[31] Nadal K.L., Whitman C.N., Davis L.S., Erazo T., Davidoff K.C. (2016). Microaggressions Toward Lesbian, Gay, Bisexual, Transgender, Queer, and Genderqueer People: A Review of the Literature. J. Sex. Res..

[32] Auguste E.E., Cruise K.R., Jimenez M.C. (2021). The Effects of Microaggressions on Depression in Young Adults of Color: Investigating the Impact of Traumatic Event Exposures and Trauma Reactions. J. Trauma. Stress.

[33] Meyer I.H. (2003). Prejudice, social stress, and mental health in lesbian, gay, and bisexual populations: Conceptual issues and research evidence. Psychol. Bull..

[34] McEwen B.S. (1998). Protective and damaging effects of stress mediators. N. Engl. J. Med..

[35] Thayer J.F., Ahs F., Fredrikson M., Sollers J.J., Wager T.D. (2012). A meta-analysis of heart rate variability and neuroimaging studies: Implications for heart rate variability as a marker of stress and health. Neurosci. Biobehav. Rev..

[36] Flentje A., Heck N.C., Brennan J.M., Meyer I.H. (2020). The relationship between minority stress and biological outcomes: A systematic review. J. Behav. Med..

[37] Flentje A., Sunder G., Tebbe E. (2025). Minority stress in relation to biological outcomes among sexual and gender minority people: A systematic review and update. J. Behav. Med..

[38] Nicholson A.A., Siegel M., Wolf J., Narikuzhy S., Roth S.L., Hatchard T., Lanius R.A., Schneider M., Lloyd C.S., McKinnon M.C. (2022). A systematic review of the neural correlates of sexual minority stress: Towards an intersectional minority mosaic framework with implications for a future research agenda. Eur. J. Psychotraumatol..

[39] Shiffman S., Stone A.A., Hufford M.R. (2008). Ecological momentary assessment. Annu. Rev. Clin. Psychol..

[40] Rosenbach H., Itzkovitch A., Gidron Y., Schonberg T. (2025). Assessing Stress Level Scores Against Wearables-Driven Physiological Measurements. Stress Health.

[41] Urueta Tapia D., Corliss H.L., Lee K.H., Calzo J.P., Jun H.-J. (2025). Assessing Minority Stress and Physiological Response Through Ecological Momentary Assessment and Sensors: Protocol for a Feasibility and Acceptability of the Stress and Heart Pilot Study. JMIR Form. Res..

[42] Williams D.R., Yu Y., Jackson J.S., Anderson N.B. (1997). Racial Differences in Physical and Mental Health: Socio-economic Status, Stress and Discrimination. J. Health Psychol..

[43] Businelle M.S., Hébert E.T., Shi D., Benson L., Kezbers K.M., Tonkin S., Piper M.E., Qian T. (2024). Investigating Best Practices for Ecological Momentary Assessment: Nationwide Factorial Experiment. J. Med. Internet Res..

[44] Metcalf O., Henry L.M., Fairbairn C.E., Flanagan J.C. (2025). Digital Technology Prediction of Anger, Aggression, and Violence: Recent Innovations and Methodological Considerations. J. Interpers. Violence.

[45] Firstbeat Technologies L. (2014). Stress and Recovery Analysis Method Based on 24-Hour Heart Rate Variability.

[46] Garmin vívosmart 4 Owner’s Manual. https://www8.garmin.com/manuals/webhelp/vivosmart4/EN-US/vivosmart_4_OM_EN-US.pdf.

[47] Quigley K.S., Gianaros P.J., Norman G.J., Jennings J.R., Berntson G.G., de Geus E.J.C. (2024). Publication guidelines for human heart rate and heart rate variability studies in psychophysiology—Part 1: Physiological underpinnings and foundations of measurement. Psychophysiology.

[48] Yang J., Kershaw K.N. (2022). Feasibility of using ecological momentary assessment and continuous heart rate monitoring to measure stress reactivity in natural settings. PLoS ONE.

[49] Ranzenhofer L.M., Engel S.G., Crosby R.D., Haigney M., Anderson M., McCaffery J.M., Tanofsky-Kraff M. (2016). Real-time assessment of heart rate variability and loss of control eating in adolescent girls: A pilot study. Int. J. Eat. Disord..

[50] Narvaez Linares N.F., Charron V., Ouimet A.J., Labelle P.R., Plamondon H. (2020). A systematic review of the Trier Social Stress Test methodology: Issues in promoting study comparison and replicable research. Neurobiol. Stress.

[51] Juarascio A.S., Crochiere R.J., Tapera T.M., Palermo M., Zhang F. (2020). Momentary changes in heart rate variability can detect risk for emotional eating episodes. Appetite.

[52] Kim J., Foo J.C., Murata T., Togo F. (2024). Reduced heart rate variability is related to fluctuations in psychological stress levels in daily life. Stress Health.

[53] Renna M.E., Shrout M.R., Madison A.A., Bennett J.M., Malarkey W.B., Emery C.F., Kiecolt-Glaser J.K. (2022). Distress disorder histories predict HRV trajectories during and after stress. Psychoneuroendocrinology.

[54] Mohammadi A., Emamgoli A., Shirinkalam M., Meftahi G.H., Yagoobi K., Hatef B. (2019). The persistent effect of acute psychosocial stress on heart rate variability. Egypt. Heart J..

[55] Roddick C.M., Seo Y.S., Barkovich S.-L., Forrester L., Chen F.S. (2025). Cardiac vagal recovery following acute psychological stress in human adults: A scoping review. Neurosci. Biobehav. Rev..

[56] Watson D., Clark L.A., Tellegen A. (1988). Development and validation of brief measures of positive and negative affect: The PANAS scales. J. Personal. Soc. Psychol..

[57] Williams D.R., Mohammed S.A. (2009). Discrimination and racial disparities in health: Evidence and needed research. J. Behav. Med..

[58] Swim J.K., Pearson N.B., Johnston K.E. (2007). Daily Encounters with Heterosexism. J. Homosex..

[59] Wright A.J., Wegner R.T. (2012). Homonegative Microaggressions and Their Impact on LGB Individuals: A Measure Validity Study. J. LGBT Issue Couns..

[60] Nadal K.L. (2013). A brief history of lesbian, gay, bisexual, and transgender people and civil rights. That’s So Gay! Microaggressions and the Lesbian, Gay, Bisexual, Transgender Community.

[61] Livingston N.A., Flentje A., Heck N.C., Szalda-Petree A., Cochran B.N. (2017). Ecological momentary assessment of daily discrimination experiences and nicotine, alcohol, and drug use among sexual and gender minority individuals. J. Consult. Clin. Psychol..

[62] Swann G., Bettin E., Ryan D.T., Clifford A., Newcomb M.E., Whitton S.W., Mustanski B. (2023). The Sexual Orientation Microaggression Inventory Short Form (SOMI-SF): Validation in Three Samples of Racially/Ethnically Diverse Sexual Minority Youth. Sex. Res. Social. Policy.

[63] Swann G., Minshew R., Newcomb M.E., Mustanski B. (2016). Validation of the Sexual Orientation Microaggression Inventory in Two Diverse Samples of LGBTQ Youth. Arch. Sex. Behav..

[64] Science of Behavior Change Ecological Momentary Assessment of Stressful Events. Open-Source Instruments and Procedures Repository. https://repository.scienceofbehaviorchange.org/.

[65] Park S.H., Petrunoff N.A., Wang N.X., van Dam R.M., Sia A., Tan C.S., Müller-Riemenschneider F. (2022). Daily park use, physical activity, and psychological stress: A study using smartphone-based ecological momentary assessment amongst a multi-ethnic asian cohort. Ment. Health Phys. Act..

[66] Littman A.J., White E., Satia J.A., Bowen D.J., Kristal A.R. (2006). Reliability and validity of 2 single-item measures of psychosocial stress. Epidemiology.

[67] Vinstrup J., Jay K., Jakobsen M.D., Andersen L.L. (2021). Single-item measures of stress during work- and private time in healthcare workers. Work.

[68] Elo A.-L., Leppänen A., Jahkola A. (2003). Validity of a single-item measure of stress symptoms. Scand. J. Work. Environ. Health.

[69] Murray A.L., Xiao Z., Zhu X., Speyer L.G., Yang Y., Brown R.H., Katus L., Eisner M., Ribeaud D. (2023). Psychometric evaluation of an adapted version of the perceived stress scale for ecological momentary assessment research. Stress Health.

[70] Stroup W.W., Milliken G.A., Claassen E.A., Wolfinger R.D. (2018). SAS for Mixed Models: Introduction and Basic Applications.

[71] Bolger N., Laurenceau J.-P. (2013). Intensive Longitudinal Methods: An Introduction to Diary and Experience Sampling Research.

[72] Dhiman P., Ma J., Qi C., Bullock G., Sergeant J.C., Riley R.D., Collins G.S. (2023). Sample size requirements are not being considered in studies developing prediction models for binary outcomes: A systematic review. BMC Med. Res. Methodol..

[73] Morris T.P., Walker A.S., Williamson E.J., White I.R. (2022). Planning a method for covariate adjustment in individually randomised trials: A practical guide. Trials.

[74] Scandola M., Tidoni E. (2024). Reliability and Feasibility of Linear Mixed Models in Fully Crossed Experimental Designs. Adv. Method Pract. Psychol. Sci..

[75] Figueroa W.S., Zoccola P.M., Manigault A.W., Hamilton K.R., Scanlin M.C., Johnson R.C. (2021). Daily stressors and diurnal cortisol among sexual and gender minority young adults. Health Psychol..

[76] Nicholas J., Bresin K. (2024). Everyday Sexual and Gender Minority Stress and Health: A Systematic Review of Experience Sampling Studies. Arch. Sex. Behav..

[77] Keenan K., Berona J., Hipwell A.E., Stepp S.D., Romito M.T. (2021). Validity of the Trier Social Stress Test in studying discrimination stress. Stress.

[78] Huebner D.M., McGarrity L.A., Perry N.S., Spivey L.A., Smith T.W. (2021). Cardiovascular and cortisol responses to experimentally-induced minority stress. Health Psychol..

[79] Seaton E.K., Zeiders K.H. (2021). Daily racial discrimination experiences, ethnic-racial identity, and diurnal cortisol patterns among Black adults. Cult. Divers. Ethn. Minor. Psychol..

[80] Cook S.H., Wood E.P., Rodrigues M., Jachero Caldas J., Delorme M. (2024). Assessment of a Daily Diary Study Including Biospecimen Collections in a Sample of Sexual and Gender Minority Young Adults: Feasibility and Acceptability Study. JMIR Form. Res..

[81] Chuntova N., Ait Abdelmalek I., Lavallée-Rodrigue K., Thériault E.-R., Hogan R., Guenoun Z., Boulette J., Romain A.J., DuBois L.Z., Juster R.-P. (2026). Intersectional stigma, health behaviors, and allostatic load among sexual and gender diverse people. Psychoneuroendocrinology.

[82] Dyar C., Herry E., Pirog S. (2024). Emotion regulation strategies and coping self-efficacy as moderators of daily associations between transgender and gender diverse (TGD) enacted stigma and affect among TGD young adults assigned female at birth. Social. Sci. Med..

[83] Smith A.U., Bostwick W.B., Burke L., Hequembourg A.L., Santuzzi A., Hughes T.L. (2023). How deep is the cut? The influence of daily microaggressions on bisexual women’s health. Psychol. Sex. Orientat. Gend. Divers..

[84] Weber J., Angerer P., Apolinario-Hagen J. (2022). Physiological reactions to acute stressors and subjective stress during daily life: A systematic review on ecological momentary assessment (EMA) studies. PLoS ONE.

[85] Vaessen T., Rintala A., Otsabryk N., Viechtbauer W., Wampers M., Claes S., Myin-Germeys I. (2021). The association between self-reported stress and cardiovascular measures in daily life: A systematic review. PLoS ONE.

[86] Rackoff G.N., Newman M.G. (2020). Reduced positive affect on days with stress exposure predicts depression, anxiety disorders, and low trait positive affect 7 years later. J. Abnorm. Psychol..

[87] Boemo T., Nieto I., Vazquez C., Sanchez-Lopez A. (2022). Relations between emotion regulation strategies and affect in daily life: A systematic review and meta-analysis of studies using ecological momentary assessments. Neurosci. Biobehav. Rev..

[88] Wardecker B.M., Surachman A., Matsick J.L., Almeida D.M. (2022). Daily Stressor Exposure and Daily Well-Being Among Sexual Minority and Heterosexual Adults in the United States: Results from the National Study of Daily Experiences (NSDE). Ann. Behav. Med..

[89] Dickerson S.S., Kemeny M.E. (2004). Acute Stressors and Cortisol Responses: A Theoretical Integration and Synthesis of Laboratory Research. Psychol. Bull..

[90] Stawski R.S., Cichy K.E., Piazza J.R., Almeida D.M. (2013). Associations among daily stressors and salivary cortisol: Findings from the National Study of Daily Experiences. Psychoneuroendocrinology.

[91] Akbar F., Mark G., Prausnitz S., Warton E.M., East J.A., Moeller M.F., Reed M.E., Lieu T.A. (2021). Physician Stress During Electronic Health Record Inbox Work: In Situ Measurement with Wearable Sensors. JMIR Med. Inform..

[92] Carreiro S., Chintha K.K., Shrestha S., Chapman B., Smelson D., Indic P. (2020). Wearable sensor-based detection of stress and craving in patients during treatment for substance use disorder: A mixed methods pilot study. Drug Alcohol Depend..

[93] Trujillo M.A., Newman D.B., Mendes W.B. (2025). Sexual orientation and daily stress and well-being. Health Psychol..

[94] Gautam K., Paudel K., Bautista B., Khati A., Sujan M.S.H., Xu R., Copenhaver M.M., Wickersham J.A., Valente P.K., Park C.L. (2026). Systematic review of ecological momentary assessment for assessing mental health among sexual and gender minorities. npj Digit. Public Health.

