# Supplementary material for: Real-Time Stress Experiences and Physiological and Psychological Responses Among LGBTQ+ Young Adults: Findings from the Stress and Heart Pilot Study
Source: Sensors (Basel). 2026 Jun 18;26(12):3872. doi: 10.3390/s26123872 (PMC13306472; doi:10.3390/s26123872)
Supplement: Supplementary file 1 [file sensors-26-03872-s001.zip › sensors-4284244-supplementary.pdf]

Table S1. Standardized effect sizes and variance explained for mixed-effects models of stress experiences and physiological and psychological outcomes.

Outcome	Predictor	Standardized β	(95% CI)	p-value	Marg. R^2	Cond. R^2
Physiological Stress Score	EDS	0.19	(−0.03, 0.42)	0.091	0.004	0.086
	SOMI-SF	0.37	(0.05, 0.69)	0.025	0.011	0.097
	EMA-SE	0.24	(−0.05, 0.52)	0.104	0.006	0.108
	CPS	0.16	(−0.06, 0.39)	0.149	0.005	0.098
	COMB	0.27	(0.07, 0.47)	0.008	0.018	0.118
Positive Affect	EDS	−0.07	(−0.19, 0.06)	0.308	0.000	0.585
	SOMI-SF	−0.12	(−0.32, 0.08)	0.231	0.001	0.502
	EMA-SE	−0.35	(−0.52, −0.18)	<.001	0.010	0.520
	CPS	−0.61	(−0.73, −0.49)	<.001	0.090	0.524
	COMB	−0.37	(−0.48, −0.26)	<.001	0.035	0.502
Negative Affect	EDS	0.31	(0.19, 0.43)	<.001	0.011	0.616
	SOMI-SF	0.12	(−0.07, 0.32)	0.221	0.001	0.540
	EMA-SE	0.59	(0.43, 0.76)	<.001	0.028	0.585
	CPS	0.68	(0.57, 0.80)	<.001	0.116	0.569
	COMB	0.51	(0.41, 0.62)	<.001	0.071	0.551

Note: Standardized β estimates, 95% confidence intervals, marginal R^2, and conditional R^2 are shown for each mixed-effects model corresponding to Table 3. Marginal R^2 represents variance explained by fixed effects only, and conditional R^2 represents variance explained by both fixed and random effects. EDS = Everyday Discrimination Scale; SOMI-SF = Adapted Sexual Orientation Microaggression Inventory–Short Form; EMA-SE = EMA of Stressful Event; CPS = Current Perceived Stress; COMB = Combined Stress Variable.

Table S2. Sensitivity analyses: Ordinal CPS and count-based COMB models compared to primary binary models

	Stress Score			Positive Affect			Positive Affect		
	β	p-value	95% CI	β	p-value	95% CI	β	p-value	95% CI
**CPS — ordinal**	1.22	0.273	(−0.96, 3.39)	−0.29	<.001	(−0.33, −0.24)	0.24	<.001	(0.21, 0.27)
**CPS — binary (primary)**	3.62	0.149	(−1.31, 8.56)	−0.52	<.001	(−0.62, −0.42)	0.44	<.001	(0.36, 0.51)
**COMB — count**	3.6	0.006	(1.03, 6.17)	−0.23	<.001	(−0.28, −0.17)	0.23	<.001	(0.19, 0.27)
**COMB — binary (primary)**	5.93	0.008	(1.53, 10.34)	−0.31	<.001	(−0.41, −0.22)	0.33	<.001	(0.26, 0.39)

Note. N = 14 participants (421 matched EMA-sensor observations for stress score; 699 for affect outcomes). Estimates are unstandardized coefficients from bivariate linear mixed-effects models. CPS = Current Perceived Stress; COMB = Combined Stress Experience. Count-based COMB was capped at 3 due to small cell sizes at 4 (n=2).

Table S3. Sensitivity analyses: Mixed-effects model estimates for physiological stress score across 30-, 60-, and 120-minute pre-EMA aggregation windows

Stress Experience Measure	Stress Score 30 min			Stress Score 60 min			Stress Score 120 min		
	β	p-value	(95% CI)	β	p-value	(95% CI)	β	p-value	(95% CI)
20 Participants (N=1001)*									
Everyday Discrimination Scale (EDS)	3.90	0.149	-(1.39 9.18)	4.06	0.091	-(0.66 8.79)	3.46	0.115	-(0.84 7.76)
14 Participants (N=699)*									
Sexual Orientation Microaggression Inventory-Short Form (SOMI-SF)	6.42	0.096	-(1.15 14.00)	8.16	0.025	(1.02 15.31)	4.09	0.207	-(2.27 10.46)
EMA of Stressful Event (EMA-SE)	5.13	0.138	-(1.66 11.92)	5.28	0.104	-(1.08 11.65)	4.76	0.096	-(0.85 10.38)
Current Stress Level (CSL)	2.93	0.274	-(2.32 8.18)	3.62	0.149	-(1.31 8.56)	1.59	0.483	-(2.85 6.03)
Combined Stress Experience (COMB)	6.27	0.009	(1.58 10.96)	5.93	0.008	(1.53 10.34)	2.85	0.157	-(1.10 6.79)

Note. Estimates are unstandardized coefficients from bivariate linear mixed-effects models. The 30-, 60-, and 120-minute columns reflect the pre-EMA aggregation window over which the physiological stress score was averaged. Physiological stress score ranged from 0 to 100.
*Sample sizes varied across predictors because of measure availability and missing sensor data.
